# Caries of the Teeth

**Published:** 1885-11

**Authors:** C. Hering


### CARIES OF THE TEETH.

*Calc. carb.* in caries of the teeth of children, particularly the scrofulous or rachitic, and if the toothache is increased by draught or cold. In caries after abuse of Mercury, *Asafœtida*, if there is a drawing pain in the jaws and copious saliva ; *Nitr. ac.*, if the teeth are loose, or feel as if they would fail out, the gums being white and swollen, and often bleeding ; *Mezereum*, the teeth decay rapidly ; if touch aggravates, and also motion.

*Phosph. ac.*, in caries of scorbutic persons—gums bleed easily, pain is worse after cold or hot, and a sensation of coldness in the roots of the molars.

*Rhus tox.*, in crusty caries, always combined with tetter, or in rheumatic or gouty patients, worse at rest, better in motion, and most at night.

*Aurum*, in secondary syphilis, or after abuse of Mercury, with looseness of teeth, ulcers in the gums, bad odor from the mouth, and heat in the head.

*China*, in carbonaceous caries, commencing with a black spot, most observed with scrofulous or tuberculous persons; if the pain is throbbing, of a congestive nature, or caused by abuse of Mercury.

*Carbo animalis*, in rending, tearing pains, caused by salt victuals, with bleeding gums and looseness of teeth, the tooth being very sensitive to the least cold.

*Lycopodium*, particularly after Calcarea, if the dull aching is worse after eating, with little tumors or ulcers on the gums.—C. Hering, in *Hom. News.*

